# COVID-19 information seeking and individuals’ protective behaviors: examining the role of information sources and information content

**DOI:** 10.1186/s12889-024-17770-0

**Published:** 2024-01-29

**Authors:** Xuefeng Zhang, Lin Du, Yelin Huang, Xiao Luo, Fenglian Wang

**Affiliations:** 1 School of Economics and Management, Anhui Polytechnic University, Wuhu, China; 2 School of Humanities, Anhui Polytechnic University, Wuhu, China

**Keywords:** COVID-19, Information sources, Information content, Individual preventive behaviors, Multiple regression analysis, FsQCA

## Abstract

**Background:**

Seeking COVID-19 information promotes individuals to adopt preventive behaviors, including wearing a mask, social distancing, staying away from risky places, and washing hands. This study aims to investigate which information and sources individuals relied on in seeking COVID-19 information and further examine their roles in individuals’ adoption of preventive behaviors.

**Methods:**

Through a statistical analysis of 1027 valid responses from citizens in different Chinese cities in 2022 to the self-designed items in an online survey, this study identified individuals’ preferred information sources and content on COVID-19. Regarding the information sources and content, the study used multiple regression analysis to examine their associations with individuals’ preventive behaviors, and further applied fuzzy-set qualitative comparative analysis (fsQCA) to explore their configurations that increase the likelihood of individuals adopting preventive behaviors.

**Results:**

Individuals preferred information about the newest prevention and control policies, precautions and treatment, and symptoms from the sources of workplace and community, social media, and social live streaming services. Additionally, individuals’ preventive behaviors were positively related to the workplace and community (*β* = 0.202, *p* <.001), social live streaming services (*β* = 0.089, *p* <.01), government department websites (*β* = 0.079, *p* <.05), television (*β* = 0.073, *p* <.05), and online news media (*β* = 0.069, *p* <.05), but were negatively associated with newspapers (*β*=-0.087, *p* <.05). Regarding information content, precautions and treatments (*β* = 0.211, *p* <.001), the newest prevention and control policies (*β* = 0.173, *p* <.001), symptoms (*β* = 0.152, *p* <.001), and official rumor-dispelling information (*β* = 0.082, *p* <.05) had a positive relationship with individuals’ preventive behaviors. In addition, fsQCA results presented eight configurations that promote individuals to adopt preventive behaviors. The total coverage and solution consistency values were 0.869 and 0.987, respectively. Furthermore, COVID-19 information content, the sources of social media and interpersonal sources, and official news media played an essential role in increasing the likelihood of individuals adopting preventive behaviors.

**Conclusions:**

Our findings demonstrated that individuals seek various COVID-19 information from multiple sources. The direct and degree of association of information sources and content with individuals’ preventive behaviors vary from source to source and from content to content. Information sources and content could combinatorially promote individuals to adopt preventive behaviors through several configurations.

## Background

On 5 May 2023, the WHO Director-General announced that COVID-19 no longer constitutes a public health emergency of international concern [1]. The pandemic would not end without efforts made by every individual in the past almost four years [2–4]. Adopting preventive behaviors, such as wearing a mask, washing hands, and social distancing, was an essential and effective effort that individuals were likely and able to make [4–6]. These behaviors contribute to preventing disease spread and protecting individuals themselves [7, 8]. So, many studies investigated what stimulates individuals to adopt the preventive behaviors, including protection motivation [9], personal values [10], emotions [11], situational motivation [6], and individual characteristics [12–14].

Seeking information on the COVID-19 virus and pandemic was also regarded as a vital approach to promoting individuals to adopt preventive behaviors [5, 6, 11, 15–17]. Individuals could access COVID-19 information from different sources, including traditional media, social media, friends, and family members [3, 18, 19]. Moreover, there were different associations between the sources where individuals acquire COVID-19 information and their adoption of preventive actions. For example, seeking information from social media facilitated individuals to exchange health information with others and search for self-protective measures [18, 20]. It thus positively correlated with individuals’ adoption of preventive actions [21, 22]. However, some studies revealed a negative relationship between information consumption on social media and individuals’ preventive behaviors [23, 24]. Besides social media, Chung and Jones-Jang [3] indicated that individuals who sought information from conservative media and Trump briefings were less likely to adopt the recommended preventive behaviors. Ng and Park [25] noted that Medicare beneficiaries who relied upon guidance from the authorities were likely to take preventive behaviors. However, those who relied on friends or family members were less likely to take preventive behaviors. Similarly, Takasaki, Coomes [26] found a negative relationship between interpersonal sources (local authorities, health workers, and neighbors/relatives) and individuals’ preventive actions. Gehrau, Fujarski [27] and Piltch-Loeb, Savoia [28] presented that searching information from traditional media, especially National TV, Newspapers, and local newspapers, increased the possibility of vaccine intention.

The above studies indicated that individuals in different locations focused on different sources for COVID-19 information. For instance, Japanese citizens most frequently used information from mass media, followed by digital media, face-to-face communication, and social media [29]. Malaysians mainly acquired information from television and internet news [23]. Social media was the most used information source in Peru [30]. Second, the information sources were related to individuals’ preventive behaviors. However, there exists a different, even converse, association for some information sources. For example, seeking information from social media may increase [21, 22] or decrease [23, 24] the likelihood of individuals adopting preventive behaviors. Third, the surveys in the above studies were conducted from 2020 to 2021. However, the utilization of information by individuals changes over time [12, 31]. In 2022, the COVID-19 pandemic was still viewed as a severe public health problem in China, and citizens still sought its related information [32, 33]. The above analysis raises the following two research questions concerning information sources during COVID-19 pandemic in 2022, China.

*RQ1a: to what extent do individuals prefer the sources to seek information related to COVID-19?*

*RQ1b: what is the association between each information source and individuals’ adoption of protective behaviors?*

On any information source, a diversity of information was presented, such as infected and recovery cases, useless preventive behaviors, and prevention regulations and policies [34, 35]. For information content on COVID, prior studies mainly concentrated on collecting and categorizing them on social media such as YouTube [36], Instagram [35], and WeChat [37] by using content analysis method. In addition, several studies used semi-structural interview and survey methods to capture what information individuals sought [18, 34, 38]. Accessing COVID-19 information increases individuals’ knowledge of COVID-19 [29, 39, 40], mitigating emotions of worry and fear, and enhancing self-efficacy of coping with COVID-19 [41, 42]. We may believe that the information content that individuals obtained is associated with their adoption of preventive behaviors. Nevertheless, to the best of our knowledge, few studies explored their relationships. Consequently, this study aims to address the following research questions concerning information content during COVID-19 pandemic in 2022, China.

*RQ2a: to what extent do individuals prefer the types of information content related to COVID-19?*

*RQ2b: what is the association between each information content and individuals’ adoption of protective behaviors?*

Individuals generally sought different information content from different sources. That is to say, information sources and content may work together to encourage individuals to adopt preventive behaviors. Prior studies separately inspected the relationship between information sources and content and individuals’ preventive behaviors. However, we have no answers to the following question that this study tries to address.

*RQ3: which information sources and content combinations promote individuals to adopt preventive behaviors?*

In summary, this study aims to reveal individuals’ preferences on the sources and content in seeking COVID-19 information in China. Furthermore, we examine the role of information sources and content in individuals’ adoption of preventive behaviors.

## Methods

### Data collection and sample

This study collected data employing an online self-reported survey through SurveyStar, a popular and widely used online platform, because of the social and physical distancing implemented during the survey. We recruited participants using a simplified snowball sampling method, where the invited participants were encouraged to post the survey on their social media accounts (e.g., WeChat and Tencent QQ) and in their virtual communities. The virtual community in this study refers to an online place for individuals in the same community to gather and interact. Before answering the questionnaires, participants were shown an information sheet that illustrates the purpose of this survey and the anonymous nature of the participation.

Our cross-sectional survey was initially expected to acquire no less than 1000 valid responses. The participants were included in meeting the criteria: the residential area in Mainland China, able to access information online. 1154 responses were received from September 23–27, 2022. Considering the 28 items in the survey, we found it difficult for participants to complete all questions carefully in twenty seconds in our pre-test. So, the individuals who answered the questions in less than twenty seconds were thought to be careless. In addition, we may think that it is impractical for an individual to pay the same attention to all the presented COVID-19 information content and information sources in the survey without any differences. As a consequence, we excluded 127 responses with a too-short response time, the same answer for all questions, and missing some answers. Finally, 1027 valid responses were retained for further analysis. The response and missing rates are 88.99% and 11.01%, respectively.

### Measures

The questionnaire consisted of the self-designed items in four parts based on the existing valid measures. Specifically, the first part gathered respondents’ demographic characteristics, including gender, age, education, and occupation [7, 18, 24]. Specifically, gender was coded as a binary variable, with 1 for male and 0 for female (reference group). The age variable was divided into seven groups: <18 (reference group), 18–25, 26–30, 31–40, 41–50, 51–60, and > 60 years. Education level was categorized into six groups: junior high school or below (reference group), high school/secondary vocational school, 2–3 years of college, undergraduate, graduate, PhD degree. Employment status was divided into eight groups: student, teacher, medical worker, employee in official sectors or state-owned companies, private company employee, self-employed, unemployed, and others (reference group).

The second part collected respondents’ preferences on the following eleven information sources [19, 23, 25, 26, 41]: social media (e.g., WeChat and Weibo), social live streaming services (e.g., TikTok and KuaiShou), online news media (e.g., TouTiao and Tencent News), websites of government departments, official online news media (e.g., People’s Daily), internet search engines (e.g., Baidu), television, radio, newspapers, friends and family members, and workplace and community. Concerning each information source, participants were asked to answer the question, “To what extent did you rely on in seeking COVID-19-related information?” A five-Likert scale ranging from 1 (Very Low) to 5 (Very High) was offered for participants to give their answers.

In the third part, participants were asked to answer, “To what extent did you pay attention to the following COVID-19 information?” This study focused on nine types of information content [13, 36–38]: evolution of the virus, symptoms, precautions and treatments, the newest prevention and control policies, the areas with a medium or high risk, the number of confirmed, symptomless, and recovery cases, negative news caused by COVID-19, positive stories of defeating COVID-19 (e.g., development of vaccines and drugs), and official rumor-dispelling information. A five-Likert scale ranging from 1 (Very Low) to 5 (Very High) was offered for participants to select for their answers.

In the final part, we presented the four preventive behaviors, i.e., wearing a mask, keeping social distance, staying away from risky places, and washing hands, that were suggested individuals adopt during the COVID-19 pandemic [4–6]. Participants expressed their opinions on each preventive behavior using a five-Likert scale where 1 = strongly disagree and 5 = strongly agree. For example, an item is “During the COVID-19 pandemic, I actively wear a mask in crowded public places”. Another item is “During the COVID-19 pandemic, I pay attention to personal hygiene, such as washing hands regularly”. In the following regression and fsQCA analysis, these four specific behaviors’ mean scores were treated as the outcome values.

### Data analysis

This study employed descriptive analysis to depict respondents’ demographic information and their preferences on the information sources and content. Further, this study explored the relationship between information sources and information content and individuals’ adoption of preventive behaviors with multiple regression analysis using IBM SPSS version 22 (IBM Corp). Finally, this study applied the fsQCA method [43] to find different configurations of information sources and content promoting individuals’ preventive behaviors. The fsQCA method was proposed by Ragin [43] that combines fuzzy-set principles with qualitative comparative analysis. In contrast to regression analysis aiming to establish the statistically significant relationship between variables, the fsQCA method offers a more comprehensive view of the relationship between the variables by uncovering various configurations of conditions that bring about the same specific outcome [44]. Furthermore, it assumes an asymmetric relationship between variables, which can address the limitation of net analysis [45].

### Common method bias

This study applied procedural and statistical control to mitigate common method bias. Specifically, following MacKenzie and Podsakoff [46]’s guidelines, the survey items were presented considering the findings in prior studies and the specific context of our study. Second, we conducted a pre-test by inviting two Ph.D. students and an associated professor in the field related to this topic and thirty citizens to fill out the questionnaires. Based on their feedback on item expression and examples of some information sources, we improved the survey to guarantee that potential respondents could capture it accurately. Furthermore, we also improved the item layout and divided the dependent and independent variables to balance the items’ order. Finally, 20 Chinese Yuan were provided to participants to attract them to participate in our survey as well as to appreciate them.

Regarding statistical control, Harman’s single-factor method was employed to test common method bias [47]. We conducted an exploratory factor analysis on the items in the survey, except for the four demographic characteristics. The results indicated that the eigenvalues of nine items were larger than 1, and the first-factor variance was 9.477% (< 40%). Moreover, we conducted a collinearity test of the data. The tolerance of dependent and independent variables was bigger than 0.3, and the variance inflation factor values were smaller than 2. The above results indicated no serious common method bias, and using multiple regression analysis is acceptable.

## Results

### Demographic characteristics of study participants

Table 1 presents demographic characteristics of the respondents. Out of 1017 respondents, 527 (55.77%) were males and 455 (44.30%) were females. Age-wise, 33.50% of respondents ranged from 26 to 30 years old, 31.84% were between 31 and 40, 24.44% were between 18 and 25, 6.04% were between 41 and 50, 2.14% were between 51 and 60, 1.66% were less than 18 years, and the rest were 60 years more. 832 (81.01%) respondents had at least a university degree. The number of respondents who had completed a junior high school or below, high school/secondary vocational school, and 2–3 years of college education level were 24, 54, and 117, respectively. Approximately two-thirds of the respondents were private company employees (*n* = 621, 60.47%). Respondents’ second and third most employment statuses were the student and official sector or state-owned company employee.

**Table 1:** Respondents’ demographic characteristics

Demographic characteristics		N	Percentage (%)
Gender	Male	572	55.70
	Female	455	44.30
Age (years)	< 18	17	1.66
	18–25	251	24.44
	26–30	344	33.50
	31–40	327	31.84
	41–50	62	6.04
	51–60	22	2.14
	> 60	4	0.39
Education	Junior high school or below	24	2.34
	High school/Secondary vocational school	54	5.26
	2–3 years of college	117	11.39
	Undergraduate	742	72.25
	Graduate	83	8.08
	PhD	7	0.68
Occupation	Student	137	13.05
	Teacher	18	1.75
	Medical worker	42	4.09
	Official sector or state-owned company employee	94	9.15
	Private company employee	621	60.47
	Self-employed	78	7.59
	Unemployed	18	1.75
	Others	22	2.14

### Individuals’ preferences on the information sources and information content

Table 2 presents the respondents’ preferences on the sources and content in seeking COVID-19 information.

**Table 2:** The respondents’ preferences on the information sources and information content

Information sources	Mean (SD)	Information content	Mean (SD)
Social media	4.03 (0.94)	Evolution of the virus	4.04 (0.80)
Social live streaming services	3.92 (1.04)	Symptoms	4.18 (0.81)
Online news media	3.73 (1.08)	Precautions and treatments	4.39 (0.79)
Government department websites	3.81 (1.10)	The newest prevention and control policies	4.48 (0.70)
Official online news media	3.45 (1.12)	The areas with a medium or high risk	4.09 (0.85)
Internet search engines	3.12 (1.09)	The number of confirmed, symptomless, and recovery cases	3.89 (0.90)
Television	3.06 (1.26)		
Radio	2.38 (1.19)		
Newspapers	2.25 (1.19)	Negative news related to COVID-19	3.84 (0.94)
Friends and family members	3.84 (0.91)	Positive stories of defeating COVID-19	4.00 (0.91)
Workplace and community	4.28 (0.71)	Official rumor-dispelling information	3.94 (0.93)

The workplace and community (*M* = 4.28, *SD* = 0.71) was the source that the respondents most relied on to seek COVID-19 information, followed by social media (*M* = 4.03, *SD* = 0.94), social live streaming services (*M* = 3.92, *SD* = 1.04), and friends and family members (*M* = 3.84, *SD* = 0.91). Newspapers (*M* = 2.25, *SD* = 1.19), radio (*M* = 2.38, *SD* = 1.19), and television (*M* = 3.06, *SD* = 1.26), were the last three sources.

Regarding information content, the newest prevention and control policies (*M* = 4.48, *SD* = 0.70), precautions and treatment (*M* = 4.39, SD = 0.79), symptoms (*M* = 4.18, SD = 0.81), medium or high risk areas (*M* = 4.09, *SD* = 0.85), and evolution of the virus (*M* = 4.04, *SD* = 0.80) were the top five types of information content that the respondents sought. The following preferred information content were positive stories of defeating COVID-19 (*M* = 4.00, *SD* = 0.91), official rumor-dispelling information (*M* = 3.94, *SD* = 0.93), the number of confirmed, symptomless, and recovery cases (*M* = 3.89, *SD* = 0.90), and negative news related to COVID-19 (*M* = 3.84, *SD* = 0.94), respectively.

### Associations between information sources and individuals’ preventive behaviors

This study applied multiple regression analysis to examine the relationship between the information sources and individuals’ preventive behaviors. The four control variables, i.e., gender, age, education, and occupation, were transformed into dummy variables in the analysis. Table 3 presents the results.

**Table 3:** Association between information sources and individuals’ preventive behaviors

Variables	B	St. Error	β	t	*p* value	95% Confidence Interval for B	
						Lower	Upper
Constant	3.292	0.187		17.603	< 0.001	2.925	3.659
Gender							
Female	Ref						
Male	-0.090	0.029	-0.093	-3.090	0.002	-0.148	-0.033
Age							
< 18	Ref						
18–25	-0.160	0.128	-0.143	-1.248	0.212	-0.410	0.091
26–30	-0.064	0.134	-0.063	-0.481	0.630	-0.327	0.198
31–40	-0.069	0.133	-0.067	-0.519	0.604	-0.329	0.191
41–50	-0.052	0.138	-0.026	-0.379	0.705	-0.323	0.218
51–60	0.062	0.160	0.019	0.389	0.697	-0.252	0.377
> 60	0.488	0.261	0.063	1.867	0.062	-0.025	1.000
Education							
Junior high school or below	Ref						
High school/secondary vocational school	0.020	0.116	0.009	0.174	0.862	-0.207	0.247
2–3 years of college	0.088	0.114	0.058	0.773	0.439	-0.136	0.312
Undergraduate	0.068	0.111	0.063	0.613	0.540	-0.150	0.285
Graduate	-0.027	0.121	-0.015	-0.222	0.825	-0.265	0.211
PhD	0.021	0.210	0.004	0.099	0.921	-0.391	0.433
Occupation							
Others	Ref						
Student	0.113	0.115	0.079	0.981	0.327	-0.113	0.340
College teacher	0.125	0.153	0.034	0.820	0.412	-0.174	0.424
Medical worker	0.179	0.128	0.074	1.397	0.163	-0.072	0.430
Official sector or state-owned company employee	0.032	0.116	0.019	0.278	0.781	-0.195	0.259
Private company employee	0.113	0.107	0.115	1.064	0.288	-0.096	0.323
Self-employed	0.036	0.114	0.020	0.313	0.754	-0.188	0.259
Unemployed	0.058	0.148	0.016	0.393	0.695	-0.232	0.348
Information sources							
Social media	0.030	0.016	0.058	1.830	0.068	-0.002	0.062
Social live streaming services	0.041	0.015	0.089	2.787	0.005	0.012	0.070
Online news media	0.031	0.015	0.069	2.013	0.044	0.001	0.061
Government department websites	0.035	0.016	0.079	2.205	0.028	0.004	0.066
Official online news media	0.020	0.016	0.048	1.303	0.193	-0.010	0.051
Internet search engines	-0.023	0.015	-0.052	-1.554	0.121	-0.052	0.006
Television	0.028	0.014	0.073	1.952	0.049	0.000	0.056
Radio	-0.016	0.017	-0.040	-0.972	0.331	-0.049	0.016
Newspapers	-0.035	0.016	-0.087	-2.216	0.027	-0.066	-0.004
Friends and family members	0.022	0.017	0.042	1.311	0.190	-0.011	0.055
Workplace and community	0.138	0.021	0.202	6.549	< 0.001	0.097	0.179
*R* ^*2*^	0.138						
*Adjusted R* ^*2*^	0.112						
*p* value	< 0.001						
*N*	1027						

Note: *** *p* <.001, ** *p* <.01, * *p* <.05. Ref: reference

There were five information sources, i.e., social live streaming services (*β* = 0.089, *p* <.01), online news media (*β* = 0.069, *p* <.05), government department websites (*β* = 0.079, *p* <.05), television (*β* = 0.073, *p* <.05), and workplace and community (*β* = 0.202, *p* <.001), that had a significantly positive relationship with individuals’ adoption of preventive behaviors. On the contrary, the source of newspapers (*β*=-0.087, *p* <.05) was negatively correlated to individuals’ preventive behaviors. The other four sources, i.e., official online news media, internet search engines, radio, and friends and family members, were not significantly associated with individuals’ preventive behaviors. Regarding coefficient scores, the workplace and community have the largest significant coefficient, followed by social live streaming services, newspapers, government department websites, television, and online news media. In terms of demographic characteristics of respondents, males (*β*=-0.093, *p* <.01) were negatively associated with their adoption of preventive behaviors compared to females. The other characteristics had no significant relationship with individuals’ preventive behaviors compared to their corresponding reference groups.

### Associations between information content and individuals’ preventive behaviors

Table 4 shows the regression results of the relationship between information content and individuals’ adoption of preventive behaviors.

**Table 4:** Association between information content and individuals’ preventive behaviors

Variables	B	St. Error	β	t	*p* value	95% Confidence Interval for B	
						Lower	Upper
Constant	2.416	0.195		12.416	< 0.001	2.034	2.798
Gender							
Female	Ref						
Male	-0.073	0.028	-0.076	-2.629	0.009	-0.128	-0.019
Age							
< 18	Ref						
18–25	-0.131	0.121	-0.117	-1.085	0.278	-0.367	0.106
26–30	-0.056	0.126	-0.055	-0.447	0.655	-0.304	0.191
31–40	-0.044	0.125	-0.042	-0.348	0.728	-0.289	0.202
41–50	0.008	0.130	0.004	0.059	0.953	-0.248	0.263
51–60	0.039	0.151	0.012	0.256	0.798	-0.258	0.336
> 60	0.472	0.247	0.061	1.910	0.056	-0.013	0.957
Education							
Junior high school or below	Ref						
High school/secondary vocational school	0.169	0.110	0.079	1.544	0.123	-0.046	0.384
2–3 years of college	0.242	0.107	0.160	2.272	0.023	0.033	0.452
Undergraduate	0.203	0.104	0.189	1.955	0.051	-0.001	0.407
Graduate	0.140	0.113	0.079	1.232	0.218	-0.083	0.362
PhD	0.361	0.200	0.062	1.806	0.071	-0.031	0.753
Occupation							
Others	Ref						
Student	0.200	0.109	0.140	1.828	0.068	-0.015	0.414
College teacher	0.190	0.144	0.052	1.315	0.189	-0.093	0.473
Medical worker	0.239	0.120	0.099	1.986	0.047	0.003	0.475
Official sector or state-owned company employee	0.132	0.109	0.079	1.211	0.226	-0.082	0.346
Private company employee	0.191	0.101	0.194	1.899	0.058	-0.006	0.388
Self-employed	0.100	0.108	0.055	0.928	0.353	-0.111	0.312
Unemployed	0.062	0.140	0.017	0.446	0.656	-0.212	0.337
Information content							
Evolution of the virus	0.027	0.020	0.045	1.333	0.183	-0.013	0.066
Symptoms	0.091	0.019	0.152	4.725	< 0.001	0.053	0.128
Precautions and treatments	0.129	0.019	0.211	6.715	< 0.001	0.091	0.167
The newest prevention and control policies	0.119	0.021	0.173	5.756	< 0.001	0.078	0.159
The areas with a medium or high risk	0.032	0.018	0.058	1.789	0.074	-0.003	0.068
The number of confirmed, symptomless, and recovery cases	0.010	0.017	0.019	0.592	0.554	-0.024	0.044
Negative news related to COVID-19	-0.017	0.015	-0.034	-1.125	0.261	-0.047	0.013
Positive stories of defeating COVID-19	-0.013	0.017	-0.025	-0.775	0.439	-0.046	0.020
Official rumor-dispelling information	0.042	0.017	0.082	2.563	0.011	0.010	0.075
*R* ^*2*^	0.225						
*Adjusted R* ^*2*^	0.204						
*p* value	< 0.001						
*N*	1027						

Note: *** *p* <.001, ** *p* <.01, * *p* <.05. Ref: reference

The results indicated that symptoms (*β* = 0.152, *p* <.001), precautions and treatments (*β* = 0.211, *p* <.001), the newest prevention and control policies (*β* = 0.173, *p* <.001), and official rumor-dispelling information (*β* = 0.082, *p* <.05) were significantly positively associated with individuals’ preventive behaviors. The other five types of information content were not significantly associated with individuals’ adoption of preventive behaviors. Furthermore, precautions and treatments and the newest prevention and control policies were the top two types of information content associated with individuals’ preventive behaviors regarding coefficient values. Regarding demographic characteristics of respondents, males (*β*=-0.076, *p* <.01) were negatively associated with individuals’ preventive behaviors compared to females. The individuals with 2–3 years of college education level (*β*=-0.160, *p* <.05) had a positive relationship between their adoption of preventive behaviors compared to those with junior high school or below education. Compared to individuals with the employment status of others, medical workers (*β* = 0.099, *p* <.05) were likely to adopt preventive behaviors. The other characteristics had no significant relationship with individuals’ preventive behaviors compared to their corresponding reference groups.

### fsQCA analysis and results

The steps for exploring the configurations of information sources and content that promote individuals’ preventive behaviors are below.

#### Step 1

Exploratory factor analysis of information sources and content.

Before applying fsQCA analysis, we first used the exploratory factor analysis method to reduce the number of antecedents. For the eleven information sources, the Cronbach’s Alpha, Kaiser-Meyer-Olkin (KMO), approximate chi-square, df, and Sig values were 0.741, 0.788, 2208.747, 66, and 0.000, respectively. For the nine types of information content, the Cronbach’s Alpha, KMO, approximate chi-square, df, and Sig values were 0.741, 0.839, 1359.328, 36, and 0.000, respectively. The above results indicated that it was acceptable for exploratory factor analysis. Table 5 shows the analysis results.

**Table 5:** Exploratory factor analysis of information sources and content

Information sources	Components			Information content	Components	
	1	2	3		1	2
Radio	0.824			Precautions and treatments	0.739	
Newspapers	0.796			Symptoms	0.668	
Television	0.746			Positive stories of defeating COVID-19	0.642	
Internet search engines	0.486			Evolution of the virus	0.561	
Official online news media		0.651		Official rumor-dispelling information	0.508	
Government department websites		0.580		Negative news related to COVID-19	0.383	
Online news media		0.532		The areas with a medium or high risk		0.809
Friends and family members			0.690	The number of confirmed, symptomless, and recovery cases		0.701
Social live streaming services			0.640			
Social media			0.638			
Workplace and community			0.611	The newest prevention and control policies		0.513

We deleted two variables, i.e., internet search engines and negative news related to COVID-19, with loadings smaller than 0.5. For the information sources, three principal components, i.e., source categories, were obtained. The first source category refers to traditional media comprising radio, newspapers, and television. The second source category involves official online news media, government department websites, and online news media. In China, the authorities generally collected and released information on COVID-19. Online news media such as Toutiao and Tencent News always quote, forward, and comment on information from official sources [48, 49]. Therefore, the information presented in these three sources generally represents the authorities’ opinions and attitudes. Consequently, this study called the second source category as official news media. The third source category comprises four specific sources, i.e., friends and family members, social live streaming services, social media, and the workplace and community. Through social media (e.g., WeChat) and social live streaming services (e.g., TikTok), an individual can acquire information from and communicate with others by using functions of liking, commenting, and sharing. An individual can also exchange information with friends, family members, colleagues, and community staff through face-to-face communication. It can be assumed that both are similar for individuals in acquiring information from others. This study categorized and named them as social media and interpersonal sources. Interpersonal sources refer to friends, family members, and individuals in the workplace and community [19, 26].

For information content, two content categories were obtained. The first category contains five kinds of information content: precautions and treatments, symptoms, positive stories of defeating COVID-19, evolution of the virus, and official rumor-dispelling information. This category is mainly about the knowledge about COVID-19. The other category includes the areas with a medium or high risk, the number of confirmed, symptomless, and recovery cases, and the newest prevention and control policies. The content in this category would be related to individuals’ decisions to travel and adopt preventive behaviors because individuals were suggested not to travel to areas with medium or high risk and take protection measures in public places. As a result, we called this content category information for prevention decisions.

#### Step 2

Calibration of data.

This study employed a direct calibration method [43] to calibrate data using fsQCA 3.0. Three reference points were set as the calibration anchors to transform data into fuzzy set membership scores. Specifically, values 1, 5, and 3 were viewed as full non-membership, full membership, and crossover point, respectively.

#### Step 3

Necessary analysis.

Table 6 shows the necessary analysis results. All conditions’ consistency and negations were less than 0.9, indicating that they were not necessary conditions promoting individuals’ preventive behaviors.

**Table 6:** Necessary analysis results

Conditions	Individuals’ preventive behaviors	
	Consistency	Coverage
Social media and interpersonal sources	0.879	0.981
~Social media and interpersonal sources	0.232	0.991
Official news media	0.817	0.982
~Official news media	0.295	0.988
Traditional media	0.466	0.987
~Traditional media	0.646	0.981
Knowledge about COVID-19	0.894	0.983
~Knowledge about COVID-19	0.217	0.982
Information for prevention decisions	0.895	0.977
~Information for prevention decisions	0.203	0.986

Note: ~ refers to logical negation

#### Step 4

Configuration analysis

Using fsQCA 3.0, we generated the truth table based on the calibration results of variables. The frequency threshold was set to 2, and the consistency threshold was 0.8 [43]. Subsequently, we conducted the configuration analysis. Table 7 presents the intermediate solutions showing sufficient information sources and content combinations that promote individuals’ preventive behaviors.

**Table 7:** fsQCA solutions

Conditions	Solutions							
	1	2	3	4	5	6	7	8
Social media and interpersonal sources	●	●	●			●	●	
Official news media	●			●		●	●	●
Traditional media	⊗	⊗	⊗	⊗	⊗			
Knowledge about COVID-19		●			●	●		●
Information for prevention decisions			●	●	●		●	●
Consistency	0.996	0.997	0.995	0.996	0.997	0.992	0.992	0.991
Raw coverage	0.533	0.581	0.581	0.534	0.584	0.747	0.741	0.762
Unique coverage	0.003	0.007	0.010	0.002	0.009	0.008	0.006	0.021
Solution coverage	0.869							
Solution consistency	0.987							

Note: ● indicates the presence of a condition (i.e., individuals prefer an information source or a type of information content more); ⊗ indicates the absence of a condition; Blank space indicates a “do not care” condition

Eight solutions could promote individuals to adopt preventive behaviors. The total coverage and solution consistency values were 0.869 and 0.987, respectively, indicating that the eight solutions in 98.7% suffice to promote individuals’ adoption of preventive behaviors, covering 86.9% of the membership scores in the outcome.

Solution 1 demonstrates that individuals were more likely to adopt preventive behaviors when preferring to seek information from social media and interpersonal sources and official news media, but not traditional media. The two categories of information content were not relevant to this solution. Solution 2 and solution 3 demonstrate that individuals were more likely to adopt preventive behaviors when preferring to seek information from social media and interpersonal sources and not from traditional media, and concerning COVID-19-related knowledge and information for prevention decisions, respectively. Solution 4 demonstrates that individuals were more likely to adopt preventive behaviors when seeking information from official news media and not from traditional media, and concerning information for prevention decisions. Solution 5 indicates that individuals were more likely to adopt preventive behaviors when preferring information about COVID-19-related knowledge and information for prevention decisions. Solutions 6 and 7 indicate that individuals were more likely to adopt preventive behaviors when seeking information from two source categories, i.e., social media and interpersonal sources and official news media, about either knowledge about COVID-19 or information for preventive decisions. Solution 8 indicates that individuals were more likely to adopt preventive behaviors when seeking information about COVID-19-related knowledge and information for prevention decisions from official news media. Solution 8, 6, and 7 are the top three solutions with the highest raw coverage, which are 0.762, 0.747, and 0.741, respectively.

## Discussion

### Information sources and information content

According to the situational theory of problem solving (STOPS) [50], seeking information is a critical action that individuals adopt to solve the problems caused by the COVID-19 virus and pandemic [5, 6, 11]. Out of nine types of information, individuals paid great attention to the information about the newest prevention and control policies, precautions and treatments, symptoms, and areas with a medium or high risk. These information offered individuals recommendations to handle their main problems, such as how to protect themselves and adjust living habits and travel and work schedual [51, 52]. It is different from what individuals were most concerned about in the early and middle stages of the pandemic, such as detailed case information [18], causes and transmission and physical health consequences of COVID-19 [34], and disease nature [38]. This result also showed that the information content that citizens were most interested in changed over time [31].

Regarding the above information, individuals were likely to search them from different sources which are complementary in ensuring the sufficiency and accuracy of acquired information [53, 54]. Our results showed that individuals sought information online and from traditional media and friends and family members. However, in terms of trustworthy of information sources [7, 19], the sources including workplace and community and government department websites received the most attention from individuals. From the perspective of convenience of acquiring information, social media and social live streaming services were used by individuals more frequently than traditional media [30, 55].

### Associations of information sources and content and individuals’ preventive behaviors

Of particular interest is the finding that the source of workplace and community had the strongest association with individuals’ preventive behaviors. One reason is that the workplace and community were the fronts of preventing virus transmission in China. The authorities required the managers or officers to deliver the newest information to every individual in time. This information was always official and accurate for individuals in making decisions to adopt recommended preventive behaviors [7, 12]. Another possible reason is that managers or officers of a workplace or community, to an extent, act as supervisors for individuals in it. Information from them would exert much influence on the individuals. Additionally, the others in the same organization likely affected an individual’s decision to adopt preventive behaviors.

Given the substantial relevance of digital technologies-based media in the time of COVID-19 pandemic [56], this study showed a significant relationship between seeking information from social live streaming services and online news media and individuals’ preventive behaviors [22, 41]. However, differing from previous studies [21–24], this study found that social media was not significantly associated with individuals’ preventive behaviors. An explanation for this may be related to our survey’s specific social media examples, i.e., WeChat and Tencent QQ. The primary function of these tools is connecting individuals and supporting them with instant messaging. In terms of communication function, they are similar to face-to-face interaction with friends, family members, and others [19, 26]. In the late pandemic stage, we have gained some recognition and knowledge about COVID-19. We knew that adopting preventive behaviors helps protect ourselves even without being reminded by others online or offline. In addition, individuals were required by Chinese authorities to adopt the necessary preventive behaviors such as wearing a mask and washing hands in public places [32, 33]. Our finding that seeking information from friends and family members did not significantly relate to individuals’ preventive behaviors supports the above analysis.

Individuals who sought information from government department websites were more likely to adopt preventive behaviors. During the COVID-19 pandemic, the authorities released the newest and high-quality information to individuals on their websites. It, on the one hand, facilitates individuals to capture adequate pandemic-related information and perceive threats and self-efficacy in time [15]. On the other hand, it mitigates individuals’ negative emotions such as worry and anxiety [12, 41]. In addition, individuals trust and recognize the quality of information from the authorities and, in turn, follow their prevention recommendations [7, 19, 57].

Four kinds of information positively correlated with individuals’ preventive behaviors. Out of them, precautions and treatments, symptoms, and the newest prevention and control policies were associated with individuals’ health, work, and life. The other significantly influential content was official rumor-dispelling information, which can help individuals capture exact information about COVID-19, reduce panic and anxiety, and refuse useless preventive behaviors [31, 40]. Furthermore, it would raise the likelihood of an individual’s adherence to suggested and effective preventive behaviors.

### The configurations of information sources and content promoting individuals’ preventive behaviors

This study revealed eight information sources and content combinations that encourage individuals to adopt preventive behaviors. Four solutions have the content category of COVID-19-related knowledge, and five contain information for prevention decisions. It shows the essential role of what information individuals seek in promoting their adoption of preventive behaviors [5, 18]. Only a solution, i.e., solution 1, does not cover any information content categories. This solution revealed a lower raw coverage and implied less empirical importance.

Regarding information sources, social media and interpersonal sources and official news media were presented in seven solutions, demonstrating the importance of seeking information from these two source categories. In addition, the results indicated a not very essential role of traditional media (i.e., newspapers, radio, and television) in stimulating individuals to adopt preventive behaviors [25, 55]. From this view, we may believe that social media and interpersonal sources and official news media increased the possibility of individuals adopting preventive behaviors more than traditional media. Furthermore, the combination of these two source categories could increase the likelihood of individuals adopting preventive behaviors. It, to an extent, can be regarded as evidence supporting the application potential of channel complementarity theory [53, 54] in individual information behaviors during health crises.

In terms of raw coverage, solution 8 has the highest raw coverage, followed by solutions 6 and 7. These three solutions share the source category of official news media. It indicates that the critical role of the authorities and their released information in promoting individuals’ preventive behaviors [7, 19, 57]. Thus, the authorities should use channels including their websites, social media, and social live streaming services to release the newest and accurate information to a broader and diverse audience. Furthermore, at least a category of information content was included in these solutions. This information helps individuals find solutions, such as individual preventive behaviors, for the COVID-19-related problems [5, 6, 51, 52].

### Comprehension of regression analysis results and fsQCA results

Two methods, i.e., multiple regression analysis and fsQCA, were applied in this study. We do not emphasize which method has an unconditional superiority because these two methods have different assumptions and research goals. The purpose of this study is to present a comprehensive description of the relationship between information sources and content and individuals’ preventive behaviors.

Multiple regression analysis results showed that individuals’ preventive behaviors were positively related to television but negatively related to newspapers and were not significantly related to radio. fsQCA results demonstrated that three of the eight solutions were irrelevant to traditional media (i.e., newspapers, radio, and television). The other five solutions indicated the absence of traditional media. Regarding the source categories of social media and interpersonal sources and official news media, multiple regression analysis results and fsQCA results are substantially similar, indicating their crucial role in promoting individuals to adopt preventive behaviors. However, there are some distinctions between these two results. For instance, social media has no significant relationship between individuals’preventive behaviors in multiple regression analysis. Nevertheless, fsQCA results indicate the important role of the combination of social media with sources of social live streaming services, friends and family members, and workplace and community in promoting individuals to adopt preventive behaviors. A possible reason for this is that social media is associated with individuals’ preventive behaviors in only a subset of cases but some cases nonetheless, making it invisible in the regression analysis [58]. In contrast, fsQCA method can identify the configurations that differ across subsets of cases. Another potential reason is that fsQCA analysis examined the source category of social media and interpersonal sources which contained social media, social social live streaming services, friends and family members, and workplace and community as a whole rather than social media in isolation.

For information content, multiple regression analysis results presented a significant relationship between individuals’ preventive behaviors and symptoms, precautions and treatments, the newest prevention and control policies, and official rumor-dispelling information. The other five types of information content had no significant association with individuals’ preventive behaviors. Using fsQCA, this study found that the two content categories, i.e., knowledge about COVID-19 and information for prevention decisions, were critical for stimulating individuals to adopt preventive behaviors.

Multiple regression analysis focused on examining the relationship between a single information source or type of information content and individuals’ preventive behaviors in this study. However, the fsQCA analysis maintained the integrity of individual case and revealed the combination or interaction of information sources and information content that increase the liklihood of individuals adopting preventive behaviors. In practice, an individual seeks various information from different sources to address problems caused by the pandemic [6, 11]. This means an individual’s decision to adopt preventive behaviors relies on both information sources and information content. As a consequence, the fsQCA results are informative and realistic. Furthermore, the fsQCA results add information to the results obtained by multiple regression analysis, which assists us in having a more comprehensive understanding of antecedents of individuals’ adoption of preventive behaviors.

### Theoretical and practical implications

The findings of this study shed light on the relationship between information sources and content and individuals’ preventive behaviors in the late stage of the COVID-19 pandemic. It would generate a comprehensive description of information seeking and individual preventive behaviors with the past studies, which mainly were conducted in the early and middle stages of the pandemic [3, 7, 18, 19, 23, 24, 27, 29, 38]. Secondly, this study prioritized the information content that individuals were concerned about and further revealed their relationship with individuals’ preventive behaviors. It contributes to the literature that mainly investigated what information individuals sought [35–37]. Thirdly, different from some studies mainly dedicated to examining the relations between information sources and individual preventive behaviors [18, 21, 23, 26, 29, 30], this study looked into information sources and content integrally and further brought light to their multiple, distinct, and equally effective combinations promoting individuals’ preventive behaviors. Additionally, the combinatorial use of fsQCA and multiple regression analysis presented a methodological contribution to the research of COVID-19 information seeking and individual’s preventive behaviors.

From a practical perspective, our findings demonstrated the positive role of health information communication in preventing the COVID-19 pandemic. Specifically, the workplace and community and government department websites were the primary sources for individuals seeking COVID-19 information. Further, seeking information from these sources positively correlated to individuals’ preventive behaviors. As a result, local official departments need to disseminate accurate and real-time information on their websites as well as to communities in order to let individuals get them in time. In addition, compared to traditional media, individuals preferred to seek information from digital technologies-based media such as social media and social live streaming services. The authorities could create a social media account to publish pandemic information quickly and conveniently [18, 41]. Regarding information content, individuals paid much attention to information about precautions and treatment, the newest prevention and control policies, and symptoms, which were different from what they were concerned about in the early stage of the pandemic [18, 34, 38]. This result, on the one hand, provides the authorities with a reference in deciding what information should be offered first to individuals. On the other hand, it requests the authorities to release information dynamically with the development of the pandemic. Finally, the fsQCA results suggested that information sources and content can increase the likelihood of individuals adopting preventive behaviors through different combinatorial paths. So, information sources and content should be comprehensively considered when disseminating pandemic information to individuals.

### Limitations

This study still suffers some limitations that provide opportunities for future research. Data in this study were collected online. Consequently, the analysis results may not represent the opinions of elders with relatively limited access to the Internet. Second, this study examined the relationship between information sources and content and individuals’ preventive behaviors but neglected the possible mediate variables (e.g., knowledge, emotions, and motivations), which could aid us in understanding the underlying mechanisms of their relationship. Finally, the small adjusted R-square values implied that some powerful antecedents of an individual’s preventive behaviors were missed in this study. Although COVID-19 no longer constitutes a public health emergency of international concern since May 2023, it did not mean died out of the COVID-19 virus. Some infected cases were still reported in some areas, which offers opportunities to increase the sample and incorporate more predictors and mediating variables in our model in future studies.

## Conclusion

This study prioritized the information sources and content that individuals sought during COVID-19 pandemic in China. Some information sources and content had a significant relationship with individuals’ preventive behaviors. Specifically, seeking information from the workplace and community had the strongest association with individuals’ preventive behaviors, followed by social live streaming services, government department websites, and online news media. In addition, four types of information, i.e., precautions and treatments, the newest prevention and control polices, symptoms, and official rumor-dispelling information, were positively associated with individuals’ preventive behaviors. Furthermore, we also presented eight configurations of information sources and content that promote individuals to adopt preventive behaviors. Our findings provide evidence-based support for understanding individuals’ information seeking behaviors and their relationship with preventive behaviors. They also offer a practical guideline for navigating individual information seeking during the COVID-19 pandemic or other possible pandemics.

## Data Availability

The datasets analyzed during this study are available from the corresponding author on reasonable request.
